# Cerebral Malaria Presenting With Shock in an Adolescent: A Case Report

**DOI:** 10.7759/cureus.29025

**Published:** 2022-09-11

**Authors:** Deborah Omoleye, Muhammad A Israr, Faria Tazin, Camille Celeste Go, Olanrewaju Saheed

**Affiliations:** 1 Division of Research, Grandville Medical Centre, Lagos, NGA; 2 Research, Larkin Community Hospital Palm Springs Campus, Hialeah, USA; 3 Internal Medicine, East Liverpool City Hospital, East Liverpool, USA; 4 Family Medicine, Larkin Community Hospital Palm Springs Campus, Hialeah, USA; 5 Internal Medicine, Federal Teaching Hospital, Ido Ekiti, NGA

**Keywords:** hematuria, infection, shock, malaria, cerebral malaria

## Abstract

Cerebral malaria (CM) is a severe infection of the brain caused by the parasite *Plasmodium falciparum*. It is commonly found as a complication of infection traveling to the brain. CM has a poor prognosis unless promptly identified and treated. This case report describes a 15-year-old girl who suddenly started experiencing a tonic-clonic seizure while playing. At the time of arrival at the emergency department, her vital signs were consistent with shock. She had hepatomegaly on physical examination, a hallmark of malarial infection due to an immune response against the proliferation of the protozoa. Peripheral blood smear for malaria parasites was positive for *P. falciparum* and *P. vivax*. The patient was started on intravenous (IV) saline, IV phenytoin, and IV metoclopramide. She was also transfused with two units of packed red blood cells. The patient was subsequently diagnosed with CM. For most patients, the course of treatment includes aggressive therapy with anti-malarial medications. She was started on broad-spectrum antibiotics and anti-malarial medications. Following two weeks of treatment, her condition improved significantly and she was discharged.

## Introduction

Cerebral malaria (CM) is the most severe pathology caused by the malarial parasite *Plasmodium falciparum *[1]. Unlike bacteria and viruses, parasite infections do not cross the blood-brain barrier. In sub-Saharan Africa, CM and acute bacterial meningitis (ABM) are the two most common causes of impaired consciousness in children [2]. Other bacterial and viral causes of meningitis among adolescents include *Neisseria meningitidis*,* Streptococcus pneumoniae*,* Enteroviruses*,* and *herpes simplex virus(HSV). Diagnosis of meningitis caused by CM is made by cerebrospinal fluid (CSF) analysis. Often, CSF cultures are negative in malaria-endemic areas regardless of the presence of low-density parasitemia [2]. Here, we present the case of a 15-year-old girl who was rushed to the emergency department (ED) after suddenly experiencing a tonic-clonic seizure. She became unresponsive to calls and touch one day before the seizure started. Her vital signs showed hypotension and tachycardia. She was pale and had cold extremities, which were consistent with shock. Catheterization was done and she presented with hematuria which cleared after a few hours. The patient was started on intravenous (IV) saline, IV quinidine, IV phenytoin, and IV metoclopramide initially. Later, carbamazepine, ciprofloxacin, clindamycin, quinine, and paracetamol were added as bacterial meningitis was suspected. A packed cell volume (PCV) test was performed on the fifth day, which showed improved hematocrit, and the patient was discharged after two weeks.

## Case presentation

A 15-year-old female resident of Nigeria was brought into the emergency room (ER) in an unconscious state by her mother. The informant was her aunt whom she lived with. She was well until three days prior when she complained of a frontal headache, and her aunt treated her with acetaminophen. Her headache worsened until she lapsed into unconsciousness, necessitating her presentation to the ER. There was no history of fever, neck pain or stiffness, jaundice or skin eruptions, fatigue, confusion, or dizziness. A few hours into admission, she developed a generalized tonic-clonic seizure lasting for five minutes.

On physical examination, the patient had a Glasgow Coma Scale (GCS) score of 6/15, she was pale with cold clammy extremities, severely dehydrated, afebrile with a temperature of 36.7°Celsius, tachycardic at 101 beats per minute, blood pressure of 90/60 mmHg, respiratory rate of 20 breaths per minute, and oxygen saturation of 99% on room air. No obvious jaundice and no enlarged lymph nodes were noted. Neither Kernig’s nor Budzinski’s signs were positive, and her pupils were equally reactive and equal in size. Abdominal examination revealed hepatomegaly, and there was no tenderness or other organ enlargement. A urethra catheter was passed for urine monitoring which drained coke-colored urine, consistent with hemoglobinuria. Other physical examinations were unremarkable. The laboratory work was significant for anemia of 7.4 g/dL, low hematocrit of 21.6%, white blood cell count of 3.85 with an elevated neutrophil count of 83.7%, lymphopenia of 12.5%, low platelet count of 63,000, and high procalcitonin of 3.46 ng/mL. The peripheral blood smear for malaria parasite was positive for *P. falciparum* and *P.* *vivax. *As shown in the* *urinalysis report in Table 1, the urine was positive for hematuria and leukocytes.

**Table 1:** Urinalysis report.

Urinalysis	Results	Reference range
Ascorbic acid	-	-
Leucocytes	++	-
Blood	+++	-
Bilirubin	+	-
Urobilinogen	+	-
Ketones	Trace	-
Nitrates	+	-
Protein	++	-
Glucose	-	-
pH	6	4.5–8
Specific gravity	1.03	1.005–1.03

Abdominopelvic ultrasound revealed hepatosplenomegaly and ascites. CSF analysis and a computed tomography (CT) scan of the brain were unremarkable. Soon after the diagnosis was made, the patient was admitted into the intensive care unit. A nasogastric tube was passed and aspirated about 900 mL of gastric content mixed with blood. She was started on IV quinine for 48 hours with alternate IV normal saline and dextrose. She was also treated with phenytoin to control the seizure activities, as well as IV paracetamol and imipenem. She was administered two units of packed red blood cells on admission.

On day two, the patient slowly regained consciousness; however, she started running a high-grade fever with a temperature of 38°Celsius. In addition, she had three episodes of non-bilious and non-projectile vomiting, but there was no associated abdominal pain. She was treated with metoclopramide and the vomiting stopped. She was thereafter transitioned to oral quinine, and clindamycin was added to her medications. A subsequent repeat of deranged laboratory values such as packed cell volume, procalcitonin, full blood count, and malaria parasite was normal.

On day 13, she did not show any signs of altered mental status and was discharged with carbamazepine and multivitamins. She was scheduled for a follow-up in two weeks.

## Discussion

The female Anopheles mosquito, a carrier of *Plasmodium *parasites, causes a life-threatening disease in humans called malaria [3]. Among the five species of the *Plasmodium* parasite, *P. falciparum* and *P. vivax* are the most life-threatening species [3]. Malaria is characterized by fever and headaches associated with anemia and hepatomegaly occasionally. Different species that cause malaria have unique fever patterns. *P. falciparum* leads to irregular fever patterns among affected patients. Malaria can be diagnosed by determining *Plasmodium* in the peripheral blood smear or bone marrow [4]. As shown in Table 2, aspartate transaminase and alanine transaminase were increased which is seen in acute malarial infection. In addition, as shown in Table 3, the abdominal ultrasound report confirmed hepatomegaly due to parasitized red blood cells being trapped in the liver.

**Table 2:** Liver function test results.

Test name	Results	Flag	Units	Reference range
Bilirubin (direct)	0.9	High	mg/dL	<0.4
Bilirubin (total) - adults	1.6	High	mg/dL	0.1–0.2
Alkaline phosphatase	329	High	U/L	64–306
Albumin	5.5	High	g/dL	3.8–5.1
Aspartate transferase	48	High	U/L	<46
Alanine transferase	44	High	U/L	<17

**Table 3:** Abdominopelvic ultrasound report.

Findings
The liver is enlarged (spans 181 mm), with a smooth, regular outline, and demonstrates homogenous parenchymal echotexture
No intra or extrahepatic ductal dilatation is seen
The vascular channels are normal
The gallbladder is normal in content and wall thickness
The spleen is enlarged (spanning 134 mm) but shows a homogeneous parenchymal echo pattern
Both kidneys are normal in size (right kidney: 105 × 31 mm; left kidney: 103 × 45 mm), shape, outline, and position, with good corticomedullary differentiation
The pelvicalyceal systems are normal
The visualized bowel loops are normal in caliber and peristaltic activity
There is free peritoneal fluid in the pelvis surrounding the uterus
An anteverted uterus is seen, measuring 49 mm (L) × 40 mm (W) × 27 mm (AP), with homogenous myometrial echotexture
The endometrial plate measures 7.8 mm in thickness
The urinary bladder is collapsed with an indwelling urethral catheter balloon
Impression: hepatosplenomegaly

CM, the most severe neurological presentation of acute falciparum malaria, is a syndrome of unconsciousness and coma [5] and presents with one to four days of fever. The incidence of seizures with CM, among which tonic-clonic seizure is the most common clinically detected seizure, is higher in children than adults [6]. The 15-year-old Nigerian patient presented to the ED with a tonic-clonic seizure along with signs of shock. Due to living in the malaria-endemic area, African children are more likely to develop severe falciparum malaria which manifests as seizures, impaired consciousness, respiratory distress, or severe anemia [7], and are more prone to developing CM.

Usually, CM results in endothelial lesions of the blood-brain barrier, and the endothelial lesions contribute to various brain injuries through sequestration of parasitized red blood cells in brain capillaries, cytokine production, immune cell/platelet accumulation, and microparticle emission [8]. Mediana et al. and Turner et al. have demonstrated that parasite-induced changes to the blood-brain barrier function can be caused by the sequestration of parasitized blood cells and host leukocytes by changes in endothelial cell intracellular signaling within cerebral microvessels [9]. This leads to inflammation of the brain, commonly seen in CM, due to endothelial damage.

Treatment of CM carries its own risk. Our patient was in the intensive care unit and was treated with quinidine. Quinidine can cause torsades de pointes related to prolonged QT intervals, as well as hypoglycemia due to hyperinsulinemia [10,11]. Frequent electrocardiography and glucose monitoring can aid the early identification, management, and prevention of these complications. Patients with a parasite load greater than 10% can benefit from exchange transfusion; however, it does not impact parasite clearance, hospital length of stay, in-hospital mortality, and/or cost of hospitalization [12]. Patients have a higher risk of death, and up to 25% of patients can develop neurological sequelae even with proper treatment [13]. Therefore, treatment must be initiated on time to maximize the chance of recovery. To avoid severe malaria-related complications, physicians must maintain a well-informed and efficient mode of treating a patient from a malaria-endemic area or with a recent travel history to an endemic region.

## Conclusions

CM is a leading cause of malaria mortality among African children. It results in endothelial lesions of the blood-brain barrier and is the most severe neurological presentation of acute falciparum malaria. It is a syndrome of unconsciousness and coma, and it usually presents with fever, anemia, hepatomegaly, and seizures. The incidence is higher in children than in adults, and* P. falciparum* and *P. vivax* are the most life-threatening species. Patients experience an increased risk of death even with appropriate treatment early in the disease course, and some patients develop neurological sequelae. Therefore, it is imperative to identify and treat CM promptly due to its poor prognosis. In this case, the patient presented in an unconscious state with a tonic-clonic seizure, and her blood smear showed both *P. falciparum* and *P.* *vivax*. She improved significantly after aggressive inpatient medical management, followed by discharge from hospital admission in stable condition. To make an early diagnosis of CM and efficiently treat the patient, clinicians should have a high index level of suspicion in patients presenting with unresolved headaches in a malaria-endemic area or patients who traveled to an endemic area. They should commence treatment as soon as possible to improve neurocognitive outcomes.
